# Investigating the effect of trigger delay on cardiac 31P MRS signals

**DOI:** 10.1038/s41598-021-87063-8

**Published:** 2021-04-29

**Authors:** Stefan Wampl, Tito Körner, Ladislav Valkovič, Siegfried Trattnig, Michael Wolzt, Martin Meyerspeer, Albrecht Ingo Schmid

**Affiliations:** 1 Medical University of Vienna, High Field MR Center, Center for Medical Physics and Biomedical Engineering, Vienna, 1090 Austria; 2 Oxford Centre for Clinical Magnetic Resonance Research (OCMR), RDM Cardiovascular Medicine, University of Oxford, Oxford, OX3 9DU United Kingdom; 3 Department of Imaging Methods, Institute of Measurement Science, Slovak Academy of Sciences, Bratislava, 814 04 Slovakia; 4 Department of Biomedical Imaging and Image-guided Therapy, Medical University of Vienna, High Field MR Center, Vienna, 1090 Austria; 5 Department of Clinical Pharmacology, Medical University of Vienna, Vienna, 1090 Austria

**Keywords:** Medical research, Metabolism, Cardiovascular biology

## Abstract

The heart’s geometry and its metabolic activity vary over the cardiac cycle. The effect of these fluctuations on phosphorus (^31^P) magnetic resonance spectroscopy (MRS) data quality and metabolite ratios was investigated. 12 healthy volunteers were measured using a 7 T MR scanner and a cardiac ^31^P-^1^H loop coil. ^31^P chemical shift imaging data were acquired untriggered and at four different times during the cardiac cycle using acoustic triggering. Signals of adenosine-triphosphate (ATP), phosphocreatine (PCr), inorganic phosphate (Pi) and 2,3-diphosphoglycerate (2,3-DPG) and their fit quality as Cramér-Rao lower bounds (CRLB) were quantified including corrections for contamination by ^31^P signals from blood, flip angle, saturation and total acquisition time. The myocardial filling factor was estimated from cine short axis views. The corrected signals of PCr and $\gamma$-ATP were higher during end-systole and lower during diastasis than in untriggered acquisitions ($P<0.05$). Signal intensities of untriggered scans were between those with triggering to end-systole and diastasis. Fit quality of PCr and $\gamma$-ATP peaks was best during end-systole when blood contamination of ATP and Pi signals was lowest. While metabolite ratios and pH remained stable over the cardiac cycle, signal amplitudes correlated strongly with myocardial voxel filling. Triggering of cardiac ^31^P MRS acquisitions improves signal amplitudes and fit quality if the trigger delay is set to end-systole. We conclude that triggering to end-systole is superior to triggering to diastasis.

## Introduction

A deranged myocardial energy metabolism is associated with many cardiovascular diseases, as heart failure or ischemic heart disease^1,2^. Phosphorus (^31^P) MRS directly measures the concentrations of the high-energy phosphates adenosine-triphosphate (ATP) and phosphocreatine (PCr) in vivo in the human myocardium. The PCr to ATP ratio has proven to be a strong indicator for cardiac function and a sensitive predictor of mortality^3,4^. ^31^P MRS further allows one to study the kinetics of cardiac metabolism in magnetization transfer experiments by measuring rates of the creatine kinase reaction which links ATP and PCr pools^5,6^.

Reproducibility studies of human cardiac ^31^P MRS at 1.5 T, 3 T and 7 T^7–9^ have shown the difficulty of establishing reliable, predictive and reproducible PCr/ATP. While these studies present various protocol optimizations and emphasize the importance of post-processing steps as saturation and blood correction, surprisingly, a systematic investigation of the effect of cardiac triggering on the quality of ^31^P MRS acquisitions has not been reported, yet.

Several recent studies did not use triggering^9–12^, supposedly with the objective to maintain saturation steady, keep measurement time at a minimum or eliminate potential complications with triggering. In cases where triggering was actually applied^8,13–20^, different approaches were pursued, reaching from immediately after the R-wave^15,16^, during diastole^8,17,18^ or approximately during systole^19,20^.

From cardiac ^1^H MRS studies on the matter^21–23^ it is known, that highest spectral quality, SNR, signal stability and repeatability are achievable when triggering to a short period around end-systole. These recommendations, however, are not necessarily directly applicable to ^31^P measurements due to several important differences, e.g. voxel size compared to myocardial thickness, acquisition duration or spectral contamination. For the same reasons correction of respiratory motion, important in ^1^H MRS, is less relevant in ^31^P MRS.

Triggered cardiac ^31^P MRS has been used to examine physiological effects of contraction on the complex dynamics of cardiac high-energy phosphates^24^. Studies on isolated, perfused and in situ animal hearts presented contradictory results on physiologic changes over the cardiac cycle^24–26^. Similar in vivo studies in humans showed no evidence of such fluctuations^27,28^, and simulations suggested that physiologic variations of cardiac high-energy phosphates are below detectability of current ^31^P-MRS techniques^29^.

Hence, the case for an optimal triggering strategy for cardiac in vivo ^31^P MRS remains. We therefore investigate the influence of cardiac triggering on ^31^P MRS data with focus on signal amplitude, spectral quality and blood contamination.

## Results

**Figure 1:**
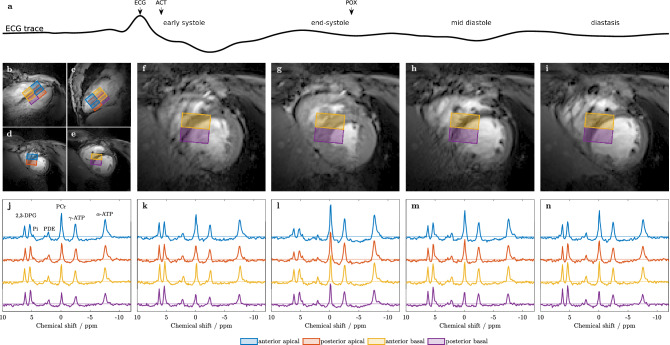
(**a**) Sample ECG trace of a single cardiac cycle depicting the trigger signals of ECG, acoustic sensor (ACT) and pulse oximeter (POX), and the four examined cardiac phases: early systole, end-systole, early diastole and diastasis. (**b**–**e**) Voxel positioning is shown on (**b**) 2-chamber, (**c**) 4-chamber, (**d**) apical short-axis and (**e**) basal short-axis cine localizer MR images. (**f**–**i**) Central short-axis cine images acquired at the four distinct cardiac phases according to the ECG trace ((**f**) early systole, (**g**) end-systole, (**h**) early diastole, (**i**) diastasis) visualize the extent of cardiac contraction. (**j**) ^31^P spectra of the untriggered and (**k**–**n**) the four triggered scans corresponding to the short-axis images (**f**–**i**) above. Voxel positions are color-encoded as depicted in the legend. Metabolite resonances of PCr, $\alpha$- and $\gamma$-ATP, 2,3-DPG and PDE are well resolved in all spectra and even the P_i_ resonance can be detected in most of the individual single-voxel spectra.

In all 14 data sets, comprising five scans each, the spectra from the four selected voxels were of good quality and fitted successfully (Fig. 1).

**Figure 2:**
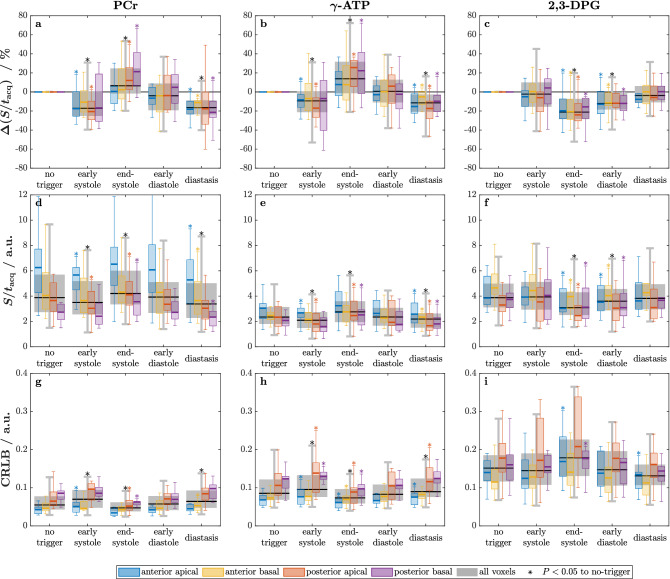
Metabolite signals $S/t_{\mathrm {acq}}$ of PCr, $\gamma$-ATP and 2,3-DPG, and the respective CRLBs over the cardiac cycle for all subjects. (**a**–**c**) Signals as deviation in % from the respective untriggered signal. (**d**–**f**) Absolute signals to visualize the influence of voxel location. (**g**–**i**) CRLBs in units of the respective signals to indicate the fitting accuracy. Boxplots depict median (line), IQR (box) and $1.5 \times \mathrm {IQR}$ (whiskers) of the study cohort; outliers not shown.

For all evaluated voxels, SNRs for PCr were $>7$, and CRLBs were $<19\,\%$. The signal amplitudes and CRLBs of PCr, $\gamma$-ATP and 2,3-DPG are shown in Fig. 2 for the voxels individually (colored boxes) and all voxels pooled (gray boxes).

In Fig. 2 the signals are shown after correction for the actual scan times ($S/t_{\mathrm {acq}}$) to provide a fair comparison between triggered and untriggered scans. While the numbers differ slightly for signals without this correction, the differences and trends are similar to $S/t_{\mathrm {acq}}$. During end-systole, these corrected metabolite signals showed a significant increase for PCr and $\gamma$-ATP, and a decrease for 2,3-DPG, with a generally more pronounced effect in posterior voxels. Diastasis consistently showed the opposite behavior. For PCr and $\gamma$-ATP the best fitting accuracy was found during end-systole while CRLBs were highest during diastasis. Selected results are presented in Table 1.

**Table 1:** Timings and results for untriggered, end-systole and diastasis triggered measurements. Data from the selected voxels of all volunteers are pooled and given as mean ± SD.

		Untriggered	End-systole	Diastasis	Mean$^{\mathrm{a}}$
Heart rate (bpm)		58 ± 9	59 ± 10	60 ± 9	60 ± 9
$\hbox {T}_{{\mathrm{R,eff}}}$ (ms)		2138 ± 217	2114 ± 358	2061 ± 318	2069 ± 303
Total scan time (min)		12.7 ± 1.5	14.1 ± 1.3	13.9 ± 1.2	13.6 ± 1.5
Trigger delay (ms)		N/A	248 ± 26	682 ± 89	N/A
Trigger delay ($\%$)		N/A	24 ± 5	68 ± 9	N/A
SNR (PCr)		23.0 ± 7.5	27.5 ± 9.6*	20.9 ± 5.8†	23.0 ± 7.2
PCr/ATP	Value	1.79 ± 0.25	1.72 ± 0.27	1.72 ± 0.33	1.75 ± 0.27
	CRLB	0.22 ± 0.07	0.17 ± 0.06*	0.22 ± 0.06†	0.21 ± 0.07
P_i_/PCr	Value	0.14 ± 0.04	0.14 ± 0.05	0.15 ± 0.07	0.15 ± 0.05
	CRLB	0.05 ± 0.01	0.05 ± 0.01	0.06 ± 0.01†	0.05 ± 0.02
pH	Value	7.050 ± 0.085	7.047 ± 0.053	7.068 ± 0.053	7.057 ± 0.061
	CRLB	0.031 ± 0.015	0.027 ± 0.011	0.035 ± 0.013†	0.031 ± 0.013

*bpm* beats per minute. $^{\mathrm{a}}$Mean of all five acquired scans per volunteer. *Significantly different to untriggered ($P<0.05$).

$^{\dagger }$ Significant difference between end-systole and diastasis ($P<0.05$).

Anterior voxels showed significantly higher PCr signals than posterior voxels, as can be seen from the absolute signals in Fig. 2. CRLBs of PCr and $\gamma$-ATP are lower in anterior voxels (Fig. 2g,h) compared to posterior voxels. The P_i_ peak area had a mean CRLB of $29 \pm 11\,\%$. No significant variations of P_i_ amplitude, chemical shift or CRLBs over the cardiac cycle were found.

**Figure 3:**
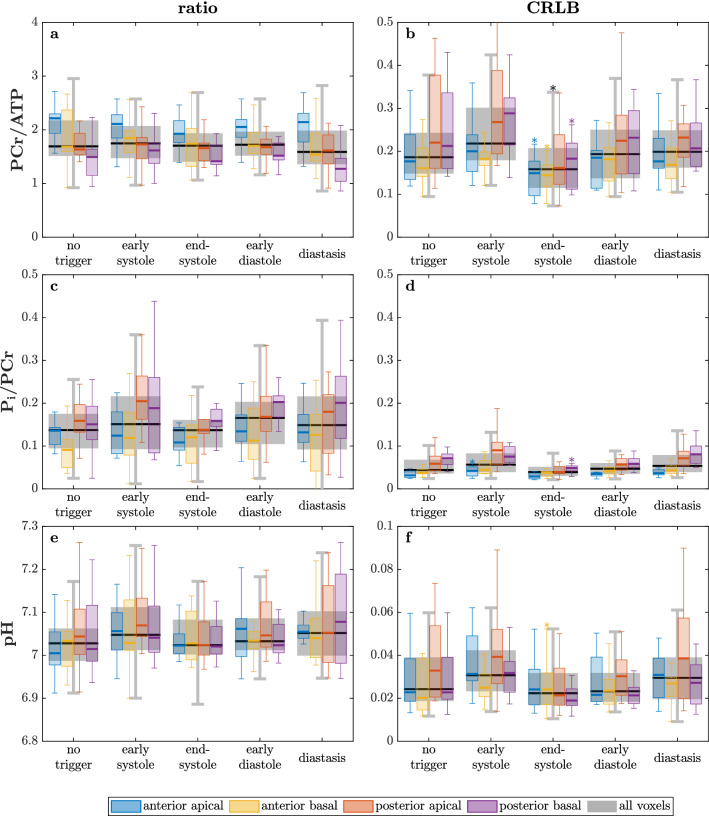
Metabolite ratios PCr/ATP (**a**), P_i_/PCr (**c**), pH (**e**) values and their respective CRLBs (**b**,**d**,**f**) over the cardiac cycle for all subjects. Boxplots depict median (line), IQR (box) and $1.5 \times \mathrm {IQR}$ (whiskers) of the study cohort; outliers not shown. *$P<0.05$ to untriggered.

Metabolite ratios PCr/ATP, P_i_/PCr and pH are presented in Fig. 3. Neither the ratios nor pH values showed significant changes with the trigger delay. The CRLBs of PCr/ATP, however, are significantly lower during end-systole. PCr/ATP is higher in anterior voxels while P_i_/PCr is higher in posterior voxels. PCr/ATP ratios (mean: $1.75 \pm 0.45$) ranged from $1.47 \pm 0.35$ in posterior-basal to $2.08 \pm 0.37$ in anterior-apical voxels (see Fig. 3a,b).

When comparing end-systole to diastasis using paired t-tests, the advantages of end-systole are clearly seen. End-systole provides significantly higher signals and lower CRLBs of the metabolites PCr and $\gamma$-ATP, while 2,3-DPG signals drop. P_i_ signals show a trend to higher values and better CRLBs during end-systole ($P=0.059$). Further, CRLBs of PCr/ATP and pH are significantly lower ($P<8 \cdot 10^{-3}$) during end-systole than during diastasis.

**Figure 4:**
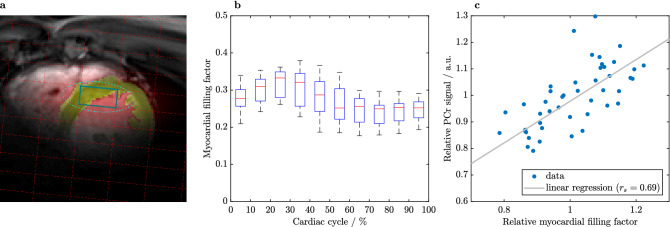
(**a**) Segmentation of left ventricular myocardium (yellow) and other tissue (red) convolved with the spatial response function (SRF) of the marked voxel (nominal: $13.75 \times 27.5\,\hbox {mm}^{2}$ in-plane). The dashed line shows the isocontour of the SRF at 64 % after filtering. The image depicts a central short axis slice during diastole with a filling factor of 29.2 % for the marked voxel. (**b**) The myocardial filling factor, i.e. myocardial volume / sensitive voxel volume, for all selected voxels and volunteers. (**c**) Correlation of the PCr signal (normalized to the untriggered signal) and the filling factor (normalized to the mean over the cardiac cycle) for all volunteers, scan times and voxels. Line depicts linear regression with $r_{\mathrm {s}} = 0.69$.

The voxel filling factor as a function of cardiac phase is depicted for all evaluated voxels in Fig. 4b. It fluctuates over the cardiac cycle with the highest filling around end-systole (20–30 % of cardiac cycle). Changes of PCr ($r_s=0.74$) (Fig. 4c), $\gamma$-ATP (0.70), 2,3-DPG ($-0.47$), P_i_ (0.43) signals and P_i_/2,3-DPG (0.55) correlated well with the filling factor (all $P<0.003$). Fitting accuracy (CRLB) of PCr, $\gamma$-ATP and PCr/ATP improved with higher filling factor ($r_s<-0.65$, $P<10^{-6}$) while it deteriorated for 2,3-DPG (0.71, $P=7 \cdot 10^{-8}$).

## Discussion

In this study, we investigated the effect of triggering on in vivo human cardiac ^31^P MR signals. Spectra from untriggered acquisitions were compared to triggered acquisitions at four different time points in the cardiac cycle. Acquiring during end-systole yields the highest signals and lowest CRLBs as compared to other cardiac phases. With our chosen parameters we further present a sound setting for reliably fitting metabolites to evaluate PCr/ATP, P_i_/PCr and pH in a clinically feasible measurement time.

We could not confirm significant variations in metabolite ratios PCr/ATP and P_i_/ATP in the myocardium over the cardiac cycle. This is in agreement with theoretical predictions^29^ and implies that results of studies which ignore cardiac motion apparently are not subject to large scale systematic errors. Nonetheless, the sensitivity of myocardial metabolite signals can be significantly improved by acquiring data during end-systole (see Fig. 2) when the heart muscle is the thickest and ventricular filling is the lowest (Fig. 4).

We conclude that voxel filling is the dominating factor when considering the effect of triggering. Although respiratory motion was not accounted for, $\sim 70\,\%$ of variance can be explained by cardiac motion, probably due to large voxel sizes. This is different in ^1^H MRS, where voxels are much smaller and typically on the order of respiratory motion. Especially the correlation of CRLBs and filling factor supports the reasonable assumption that the quality of spectral fitting improves with a higher myocardial voxel filling.

We observed a bigger effect of triggering in posterior voxels, which suggests that the anterior ones have some contribution originating from skeletal muscle signals. This is in line with higher PCr/ATP ratios in these voxels. Therefore higher resolution in the anterior-posterior direction or other methods to improve the spatial response function should be used.

In contrast to our results, two prior studies^27,28^ reported no significant differences between metabolite concentrations of diastolic and systolic spectra. In our study, however, a smaller nominal voxel size was used. This reduces potential contamination from skeletal muscle but accentuates the susceptibility to partial filling of the voxel, a fact already acknowledged by Grist et al.^27^. Increased SNR at 7 T presumably contributed to our finding.

For cardiac ^1^H MRS the optimal trigger delay was found at mid- to end-systole^23^, which agrees well with our findings for ^31^P MRS. Results of ^1^H MRS are, however, not directly applicable to ^31^P MRS as they differ in several important aspects. In ^1^H MRS, the predominant water signal is as much in blood as in tissue, small fluctuations in shim and frequencies can lead to severe artifacts. Therefore voxels are much smaller (also due to SNR) which are therefore affected considerably more by motion.

While diastasis offers the longest quiescent period with relatively little cardiac motion, its onset shows a strong dependence on the heart rate which is a liability in long experiments such as ^31^P MRS. End-systole, however, provides smaller inter- and intra-subject variability, and is much less affected by the heart-rate^30^. This is particularly relevant if the heart rate is expected to change, e.g., during a long scan or under increased work-load (i.e. stress), hence making end-systole even more favourable for triggering to.

Cardiac motion induced variance can be largely explained by voxel filling, a main limitation of this study lies in the lack of respiratory motion correction. For multi-nuclear sequences currently no robust methods are available by default. Application of respiratory belts can be challenging^31^, as is the implementation of image-based navigators^32^, and only few successful approaches have been reported on the matter^20^. In our specific case, typical respiratory displacements^33^ are smaller than the effective voxel (indicated by the shaded area in Fig. 4a). Even if respiratory motion somewhat obscured the effect of triggering, our results provide conclusive evidence for improvements certainly attributable to triggering. With smaller voxels the correction of breathing motion can substantially improve spectra, as known from ^1^H MRS^22^.

Since our signals are normalized to the total acquisition times and the flip angle was lower than the Ernst angle, the potential gain through triggering is underestimated. The SNR of ATP and PCr are especially relevant in kinetic studies, i.e. magnetization transfer measurements of the creatine kinase reaction, and our results are directly applicable there.

Spectral contamination due to signal arising from blood heavily affects the accuracy of cardiac ^31^P data. Blood 2,3-DPG is overlapping the small P_i_ peak in ^31^P spectra. Blood also contains ATP and P_i_, adding to their quantification uncertainty proportional to the 2,3-DPG signal. Hence, reducing 2,3-DPG signals directly, as achieved by triggering to end-systole, increases the reliability of the ATP and P_i_ signals.

In most voxels P_i_ was visible at least as a shoulder to 2,3-DPG and quantifiable. P_i_ quantification and hence pH calculation accuracy are limited by the relatively high CRLBs. In particular the chemical shift of P_i_ could be best fitted end-systole, while CRLBs were negatively affected by triggering to diastasis. This demonstrates that even at shorter scan and repetition times than previously used^12^, it can be measured by 7 T ^31^P MRS, provided that $\mathrm{B}_0$ shimming is done.

We conclude, that triggering of cardiac ^31^P MRS and the selected cardiac phase have a substantial effect on data quality and signal yield. Cardiac ^31^P MRS without triggering is feasible, however, best signals and CRLBs are achieved with triggering to end-systole.

## Methods

The study was approved by the ethics committee of the Medical University of Vienna, Austria (EK Nr. 1670/2017) and conducted according to the Declaration of Helsinki in its latest version. Informed consent was obtained from all participants. Twelve healthy subjects (6f / 6m, age 21–39 years, body mass index 20.7–25.2 $\,\hbox {kg}/\hbox {m}^{2}$) were studied of which two were scanned twice. The measurements were performed in supine position on a Magnetom 7 T MR scanner (Siemens, Erlangen, Germany) using a cardiac ^31^P loop ($d=14\,\hbox {cm}$) and $14 \times 22\,\hbox {cm}^{2}$ ^1^H surface coil (Rapid Biomedical, Rimpar, Germany).

Subjects were equipped with an acoustic triggering device (Mri.Tools GmbH, Berlin, Germany) for synchronizing MR acquisitions, and a pulse oximeter as backup. In case of two volunteers with unreliable acoustic trigger signal, the pulse oximeter was required for online triggering. In these cases, trigger delays were set accordingly to compensate the temporal delay between pulse and acoustic trigger (see Fig. 1a)^34^. Cardiac localizer views were acquired during exhaled breath-hold state using a retrospectively gated 2D cine FLASH (Fig. 1b–i). Per subject second order $\mathrm{B}_0$ shimming was performed before the first ^31^P scan using coronal dual-echo GRE fieldmap scans with the shim volume covering the whole heart. The triggered (no delay) and breath-hold (exhaled) shim scans were repeated several times until shim currents converged.

Acquisition weighted (3 averages) pulse-acquire chemical shift imaging (CSI)^35,36^ was used to acquire localized cardiac ^31^P spectra (Fig. 1j–n) with the following settings: receiver bandwidth 6 kHz and 512 complex samples; sampling duration 85 ms; nominal spatial resolution $8 \times 16 \times 8$ with Hamming filtering; voxel sizes: $27.5\times 13.75\times 25\,\hbox {mm}^{3}$ (9.5 ml). The CSI volumes were aligned to the heart’s long-axis, the septum and with the highest resolution parallel to the chest wall, to minimize contamination from skeletal muscle. The CSI measurements were repeated in each subject, with one untriggered and four acoustically triggered acquisitions. Trigger delays were identified visually on the cine images reconstructed to 20 cardiac phases and equidistantly distributed between early systole and end-diastole. Cine images were reacquired between CSI scans to confirm trigger delays for the selected cardiac phases. In most volunteers slightly increased heart-rates ($9\pm 12\,\%$) were observed during their last compared to the first CSI scan. In most cases the drift was smaller than the temporal resolution of the cine images. This did therefore neither affect the selected trigger delay nor substantially reduce the effective repetition time $\hbox {T}_{{\mathrm{R,eff}}}$ (see Table 1).

Every second trigger signal was used to start ^31^P CSI acquisitions, resulting in a $\hbox {T}_{\mathrm{R}}$ of approximately 2 s (see also Table 1). In untriggered acquisitions, $\hbox {T}_{\mathrm{R}}$ was set individually to two R-R intervals. The seemingly odd choice for the repetition time was selected as a compromise between efficiency and visibility of inorganic phosphate (P_i_). Our lower flip angle weighs the signal favorably to long $\hbox {T}_{1}$ P_i_ in contrast to 2,3-DPG from flowing blood. The total measurement time was dependent on the individual heart rate, resulting in $12.7 \pm 1.5\,\hbox {min}$ for untriggered and $13.9 \pm 1.4\,\hbox {min}$ ($P=0.005$) for triggered scans, respectively. The whole scan sessions took between 105 min and 115 min.

For analysis, four voxels per subject were selected as shown in Fig. 1b–i, with the positions referred to as: anterior apical, posterior apical, anterior basal and posterior basal. Voxel-wise flip angle estimations were determined using an external reference, as described previously^10^.

In total 280 spectra were analyzed using AMARES as implemented in the OXSA toolbox^37^, fitting the following metabolites as Lorentzian lines: PCr, $\alpha$- and $\gamma$-ATP doublets, two phosphodiester resonances of glycerophosphocholine and glycerophosphoethanolamine, two 2,3-DPG peaks as singlet (3-DPG) and doublet (2-DPG)^38^ and inorganic phosphate (P_i_). To improve the convergence of the fit for P_i_, linewidth and phases were locked within two groups: (i) PCr and P_i_ and (ii) phosphodiesters. Additionally, the phases of the 2,3-DPG peaks were locked to the phase of the PCr peak. Correction of ATP and P_i_ signals for contribution from blood was based on the 2,3-DPG signals ($\gamma$-ATP/2,3-DPG = 0.279 and P_i_/2,3-DPG = 0.070^12,39^), followed by saturation correction using 7 T ^31^P $\hbox {T}_{1}$ relaxation times^10,12^. For saturation correction, the effective repetition time $\hbox {T}_{{\mathrm{R,eff}}}$ was estimated as the median of the duration between readouts. To account for potentially longer acquisition time ($t_{\mathrm {acq}}$) during triggered scans, peak amplitudes (*S*) were compared using signal per unit time ($S/t_{\mathrm {acq}}$).

SNR was determined as the peak height over the baseline standard deviation after application of a matched filter^10^. Changes in signal can be considered equivalent to changes in SNR as long as the noise characteristics are the same between trigger times. When comparing signals acquired with different trigger delay and from different voxels, we used signal amplitudes since the limited number of noise samples introduced additional variance to SNR. Cramér-Rao lower bounds (CRLBs)^40^ were used to determine uncertainty of metabolite concentrations and ratios. PCr/ATP ratios were calculated using $\gamma$-ATP, and P_i_ fits with CRLB $>50\,\%$ were excluded. pH was derived from a modified Henderson-Hasselbalch relationship^41^.

Two-way analysis of variance (ANOVA) was employed to check for effects of trigger delay and voxel position on the spectral parameters of the metabolites PCr, $\gamma$-ATP, 2,3-DPG and P_i_, and their respective ratios. The trigger delays providing minimal tissue velocities induced by cardiac contraction are the two quiescent phases in the cardiac cycle during end-systole and diastasis, as explored for cardiac MR angiography^42^. Hence, pairwise differences between untriggered, end-systole and diastasis data were examined during post-hoc tests (Bonferroni corrected paired Student’s t-tests).

The left ventricle was drawn manually on a stack of short-axis cine images with ten phases per cardiac cycle. The generated mask convolved with the spatial response function delivered the myocardial filling factor for each of the selected CSI voxels. Spearman’s rank correlation coefficient $r_s$ of the filling factor and the metabolite signals, ratios and CRLBs was evaluated.

## References

[1] Bizino MB, Hammer S, Lamb HJ (2014). Metabolic imaging of the human heart: Clinical application of magnetic resonance spectroscopy. Heart.

[2] Taegtmeyer H (2004). Cardiac metabolism as a target for the treatment of heart failure. Circulation.

[3] Neubauer S (2007). The failing heart—An engine out of fuel. N. Engl. J. Med..

[4] Ingwall JS (2002). Is creatine kinase a target for AMP-activated protein kinase in the heart?. J. Mol. Cell. Cardiol..

[5] Bashir A, Gropler R (2014). Reproducibility of creatine kinase reaction kinetics in human heart: A 31P time-dependent saturation transfer spectroscopy study. NMR Biomed..

[6] Clarke WT, Robson MD, Neubauer S, Rodgers CT (2017). Creatine kinase rate constant in the human heart measured with 3D-localization at 7 tesla. Magn. Reson. Med..

[7] Lamb HJ (1996). Reproducibility of human cardiac31P-NMR spectroscopy. NMR Biomed..

[8] Tyler DJ (2009). Reproducibility of 31P cardiac magnetic resonance spectroscopy at 3 T. NMR Biomed..

[9] Ellis J, Valkovič L, Purvis LA, Clarke WT, Rodgers CT (2019). Reproducibility of human cardiac phosphorus MRS (31P-MRS) at 7 T. NMR Biomed..

[10] Rodgers CT (2014). Human cardiac 31P magnetic resonance spectroscopy at 7 tesla. Magn. Reson. Med..

[11] Dass S (2015). Exacerbation of cardiac energetic impairment during exercise in hypertrophic cardiomyopathy: A potential mechanism for diastolic dysfunction. Eur. Heart J..

[12] Valkovič L (2019). Measuring inorganic phosphate and intracellular pH in the healthy and hypertrophic cardiomyopathy hearts by in vivo 7T 31P-cardiovascular magnetic resonance spectroscopy. J. Cardiovasc. Magn. Reson..

[13] von Kienlin M (2001). Advances in human cardiac 31P-MR spectroscopy: SLOOP and clinical applications. J. Magn. Reson. Imaging.

[14] Bakermans AJ (2017). Human cardiac 31P-MR spectroscopy at 3 tesla cannot detect failing myocardial energy homeostasis during exercise. Front. Physiol..

[15] Köstler H (2006). Age and gender dependence of human cardiac phosphorus metabolites determined by SLOOP31P MR spectroscopy. Magn. Reson. Med..

[16] Apps AP (2020). Quantifying the effect of dobutamine stress on myocardial Pi and pH in healthy volunteers; a 31P MRS study at 7T. Magn. Reson. Med..

[17] Shivu GN (2010). 31P magnetic resonance spectroscopy to measure in vivo cardiac energetics in normal myocardium and hypertrophic cardiomyopathy: Experiences at 3T. Eur. J. Radiol..

[18] Clarke WT (2019). Localized rest and stress human cardiac creatine kinase reaction kinetics at 3 T. NMR Biomed..

[19] Masuda Y (1992). High-energy phosphate metabolism of the myocardium in normal subjects and patients with various cardiomyopathies. The study using ECG gated MR spectroscopy with a localization technique. Jpn. Circ. J..

[20] Kozerke S, Schär M, Lamb HJ, Boesiger P (2002). Volume tracking cardiac 31P spectroscopy. Magn. Reson. Med..

[21] Weiss K, Summermatter S, Stoeck CT, Kozerke S (2014). Compensation of signal loss due to cardiac motion in point-resolved spectroscopy of the heart. Magn. Reson. Med..

[22] de Heer P, Bizino MB, Lamb HJ, Webb AG (2016). Parameter optimization for reproducible cardiac 1H-MR spectroscopy at 3 Tesla. J. Magn. Reson. Imaging.

[23] Carlsson Å, Sohlin MC, Lagerstrand KM, Ljungberg M, Aronsson EF (2017). The influence of cardiac triggering time and an optimization strategy for improved cardiac MR spectroscopy. Z. Med. Phys..

[24] Spindler M (2001). Temporal fluctuations of myocardial high-energy phosphate metabolites with the cardiac cycle. Basic Res. Cardiol..

[25] Fossel ET, Morgan HE, Ingwall JS (1980). Measurement of changes in high-energy phosphates in the cardiac cycle using gated 31P nuclear magnetic renonance. Proc. Natl. Acad. Sci..

[26] Balaban R, Heineman F (1989). Control of mitochondrial respiration in the heart in vivo. Mol. Cell Biochem..

[27] Grist TM (1989). Gated cardiac MR imaging and P-31 MR spectroscopy in humans at 1.5 T. Work in progress. Radiology.

[28] Mitsunami K (1992). In vivo 31P nuclear magnetic resonance spectroscopy in patients with old myocardial infarction. Jpn. Circ. J..

[29] Weiss K, Bottomley PA, Weiss RG (2015). On the theoretical limits of detecting cyclic changes in cardiac high-energy phosphates and creatine kinase reaction kinetics using in vivo 31P MRS. NMR Biomed..

[30] Slavin GS, Fung M (2014). Electromechanical analysis of optimal trigger delays for cardiac MRI. J. Cardiovasc. Magn. Reson..

[31] Santelli C (2011). Respiratory bellows revisited for motion compensation: Preliminary experience for cardiovascular MR. Magn. Reson. Med..

[32] Wampl S (2020). Cardiac 31P MR spectroscopy with interleaved 1H image navigation for prospective respiratory motion compensation—initial results. Proc. Intl. Soc. Mag. Reson. Med..

[33] Wang Y, Riederer SJ, Ehman RL (1995). Respiratory motion of the heart: Kinematics and the implications for the spatial resolution in coronary imaging. Magn. Reson. Med..

[34] Frauenrath T (2010). Acoustic cardiac triggering: A practical solution for synchronization and gating of cardiovascular magnetic resonance at 7 Tesla. J. Cardiovasc. Magn. Reson..

[35] Pohmann R, Von Kienlin M (2001). Accurate phosphorus metabolite images of the human heart by 3D acquisition-weighted CSI. Magn. Reson. Med..

[36] Robson MD, Tyler DJ, Neubauer S (2005). Ultrashort TE chemical shift imaging (UTE-CSI). Magn. Reson. Med..

[37] Purvis LAB (2017). OXSA: An open-source magnetic resonance spectroscopy analysis toolbox in MATLAB. PLoS ONE.

[38] Schmidt O, Bunse M, Dietze GJ, Lutz O, Jung WI (2001). Unveiling extracellular inorganic phosphate signals from blood in human cardiac 31P NMR spectra. J. Cardiovasc. Magn. Reson..

[39] Horn M, Kadgien M, Schnackerz K, Neubauer S (2000). Spectroscopy: 31P-nuclear magnetic resonance spectroscopy of blood: A species comparison. J. Cardiovasc. Magn. Reson..

[40] Cavassila S, Deval S, Huegen C, van Ormondt D, Graveron-Demilly D (2001). Cramér-Rao bounds: An evaluation tool for quantitation. NMR Biomed..

[41] de Graaf RA (2007). In Vivo NMR Spectroscopy.

[42] Lu B (2001). Coronary artery motion during the cardiac cycle and optimal ECG triggering for coronary artery imaging. Invest. Radiol..

